# Bilateral dilated nonreactive pupils secondary to rocuronium infusion in an ARDS patient treated with ECMO therapy: A case report

**DOI:** 10.1097/MD.0000000000021819

**Published:** 2020-08-21

**Authors:** Huaiwu He, Zhaoxia Yu, Jiahui Zhang, Wei Cheng, Yun Long, Xiang Zhou, Siyi Yuan

**Affiliations:** a Department of Critical Care Medicine, Peking Union Medical College Hospital, Chinese Academy of Medical Sciences, Dongcheng District, Beijing; b Department of Critical Care Medicine, The First Affiliated Hospital of Xinjiang Medical University, Urumqi, Xinjiang Province, China.

**Keywords:** ARDS, dilated pupils, ECMO, rocuronium

## Abstract

**Rationale:**

Pupil monitoring for neurologic examination has become a regular clinical practice during extracorporeal membrane oxygenation (ECMO) therapy. Sudden dilation of pupils always indicates a severe cerebrovascular event. However, bilateral dilated nonreactive pupils secondary to neuromuscular blockade are uncommon and widely ignored in adult acute respiratory distress syndrome (ARDS) patients. This is the first case report of bilateral dilated nonreactive pupils caused by rocuronium in an ARDS patient receiving ECMO treatment.

**Patient concerns:**

Bilateral dilated nonreactive pupils were found in an ARDS patient who received V-V ECMO therapy. However, CT angiography did not indicate the occurrence of a cerebrovascular event. Drugs that could potentially result in dilated nonreactive pupils were checked.

**Diagnosis:**

Bilateral dilated nonreactive pupils were caused by rocuronium infusion.

**Interventions:**

Rocuronium infusion was stopped.

**Outcomes:**

Bilateral dilated nonreactive pupils were resolved 20 h after rocuronium infusion was stopped.

**Lessons:**

Neuromuscular blockade should be taken into consideration when bilateral dilated nonreactive pupils are found in ARDS patients treated with ECMO therapy.

## 1 Introduction

Intracranial hemorrhage is a severe complication during extracorporeal membrane oxygenation (ECMO) therapy with anticoagulation.^[1]^ Monitoring of the pupils is commonly performed during neurologic examination and has become a clinical guideline during ECMO therapy. Sudden dilation of pupils always indicates a severe cerebrovascular event that requires a rapid diagnosis.

A neuromuscular-blocking agent was always administered by continuous intravenous infusion early in the course of acute respiratory distress syndrome (ARDS) for patients with a PaO_2_/FiO_2_ <150.^[2]^ However, bilateral dilated nonreactive pupils secondary to neuromuscular blockade have not been reported in ARDS patients treated with ECMO therapy.

Here, we report a case of reversible fixed and dilated pupils caused by rocuronium infusion in an ARDS adult patient treated with ECMO and review the literature on cases of neuromuscular-blocking agents resulting in dilated pupils in clinical practice.

## 2 Case description

A female patient was transferred to our hospital ICU department because of ARDS and sepsis (H1N1 infection and bacterial pneumonia). She had received mechanical ventilation 2 weeks before in the local hospital. Moreover, the patient had been in high mechanical ventilation conditions (FiO_2_ 100% and PEEP 15 cm H_2_O), but hypoxemia was persistent (SaO_2_ 70–80%). Hence, the patient was undergoing V-V ECMO therapy. A combination of remifentanil, midazolam, and propofol infusion was used for sedation and analgesia. For lung protection, rocuronium was administered with the aim of controlling spontaneous breathing on day 1 of ECMO therapy.

The patient's pupils were 2 mm and sluggishly reactive under sedation, and the pupils were monitored every 4 h during the periods of ECMO treatment. Heparin was continuously infused for anticoagulation, and APTT was maintained at ∼40 s. The pupils were noted to be 6 mm and nonreactive when rocuronium was infused for 79 h at a dose of 20 mg/h. A severe cerebrovascular event was suspected. Head computed tomography revealed no evidence of edema, mass effect, or hemorrhage. Moreover, CT angiography also did not find obstruction of cerebral vessels. Other drugs potentially resulting in pupil dilation, such as atropine, had not been used. Hence, the effect of rocuronium on dilated nonreactive pupils was suspected. After 4 h of the discontinuation of rocuronium, the pupils started to decrease in size. Furthermore, the size of pupils decreased to 2 mm, and the pupils became reactive to light 20 h after stopping rocuronium infusion. The patient died ∼3 weeks later from progressive infection and lung failure.

## 3 Literature review

A systematic literature search was conducted in PubMed on articles in English using the following search terms: “dilated pupils” and “neuromuscular block” or “atracurium” or “vecuronium” or “rocuronium.” After selection, only 2 articles reported that neuromuscular blocking agents caused dilated pupils.^[3,4]^ Related reports are rare in the published literature, and this was the first case of dilated pupils caused by rocuronium in an ARDS patient receiving ECMO therapy.

## 4 Discussion

In patients undergoing mechanical ventilation for treatment of ARDS, neuromuscular blocking agents may improve oxygenation and decrease ventilator-induced lung injury.^[5]^ Moreover, ECMO functions as a salvage therapy for refractory hypoxemia caused by ARDS, and neuromuscular blocking agents are more commonly used at the early stage.

Since anticoagulation is used to prevent membrane oxygenator thrombosis, ARDS patients receiving ECMO treatment are at a high risk of intracranial hemorrhage. Pupil monitoring has been used as an important and easy method to identify early complications of anticoagulation during ECMO therapy at the bedside. The present case indicated that neuromuscular blockade could confound neurologic examination via pupil monitoring in ARDS patients treated with ECMO therapy. Moreover, neuromuscular blockade is widely used in the management of critically ill and injured patients.^[6]^ Studies have reported that long-term infusion of neuromuscular blockade agents causes reversed dilated pupils. Schmidt et al reported that atracurium or vecuronium infusion caused dilated and nonreactive pupils in 3 adolescent ARDS patients.^[3]^ Recently, Joyce C found that rocuronium resulted in bilateral fixed and dilated pupils in a 1-week-old low-birth-weight neonate.^[4]^

Rocuronium is highly ionized and has relatively low lipophilicity as a nondepolarizing neuromuscular blocking drug (NMBD). Theoretically, rocuronium as a nondepolarizing neuromuscular blocking agent could not cross the blood–brain barrier and might not be considered a causative agent for fixed mydriasis. However, rocuronium may have crossed the disturbed blood–brain barrier and disrupted central cholinergic transmission in the present case. Inflammation and oxidative stress play an important role in sepsis-associated encephalopathy.^[7]^ Recently, Ortega et al reported that pediatric ECMO patients with acquired brain injury exhibited the induction of proinflammatory cell signaling, the robust activation of adaptive immune cells, and CNS-targeted adaptive immune responses.^[8]^ Hence, we speculated that inflammation and oxidative stress might have impaired the blood–brain barrier in the ARDS patient with sepsis described in this study. Importantly, the dilated pupils recovered after stopping the infusion of rocuronium. Hence, it was reasonable that continuous rocuronium infusion resulted in fixed mydriasis in this case.

In summary, clinicians should pay careful attention to cases in which nondepolarizing neuromuscular blocking agents cause fixed and dilated pupils in ARDS patients treated with ECMO therapy.

## Author contributions

**Conceptualization:** Huaiwu He, Zhaoxia Yu, Jiahui Zhang, Wei cheng, Yun Long, Xiang Zhou, Siyi Yuan.

**Data curation:** Huaiwu He, Jiahui Zhang.

**Resources:** Xiang Zhou.

**Software:** Yun Long.

**Visualization:** Zhaoxia Yu, Jiahui Zhang, Wei cheng.

**Writing – original draft:** Huaiwu He, Zhaoxia Yu, Jiahui Zhang, Wei cheng, Yun Long, Xiang Zhou, Siyi Yuan.

**Writing – review & editing:** Huaiwu He, Zhaoxia Yu, Jiahui Zhang, Wei cheng, Yun Long, Xiang Zhou, Siyi Yuan.

## References

[1] Luyt CE Bréchot N Demondion P Brain injury during veno venous extracorporeal membrane oxygenation. Intensive Care Med 2016;42:897–907. 27007107 10.1007/s00134-016-4318-3

[2] Murray MJ DeBlock H Erstad B Clinical practice guidelines for sustained neuromuscular blockade in the adult critically ill patient. Crit Care Med 2016;44:2079–103. 27755068 10.1097/CCM.0000000000002027

[3] Joyce C Greenwald BM Han P Bilateral dilated nonreactive pupils in a neonate after surgery. A A Case Rep 2016;6:286–7. 27002754 10.1213/XAA.0000000000000306

[4] Schmidt JE Tamburro RF Hoffman GM Dilated nonreactive pupils secondary to neuromuscular blockade. Anesthesiology 2000;92:1476–80. 10781295 10.1097/00000542-200005000-00039

[5] Papazian L Forel JM Gacouin A ACURASYS Study Investigators. Neuromuscular blockers in early acute respiratory distress syndrome. N Engl J Med 2010;363:1107–16. 20843245 10.1056/NEJMoa1005372

[6] Stöckl M Testori C Sterz F Continuous versus intermittent neuromuscular blockade in patients during targeted temperature management after resuscitation from cardiac arrest—a randomized, double blinded, double dummy, clinical trial. Resuscitation 2017;120:14–9. 28860012 10.1016/j.resuscitation.2017.08.238

[7] Kuperberg SJ Wadgaonkar R Sepsis-associated encephalopathy: the blood-brain barrier and the sphingolipid rheostat. Front Immunol 2017;8:597 eCollection 2017. 28670310 10.3389/fimmu.2017.00597 PMC5472697

[8] Ortega SB Pandiyan P Windsor J A pilot study identifying brain-targeting adaptive immunity in pediatric extracorporeal membrane oxygenation patients with acquired brain injury. Crit Care Med 2019;47:e206–13. 30640221 10.1097/CCM.0000000000003621 PMC6377324

